# Supplementary material for: Comparative Pathology of Domestic Pigs and Wild Boar Infected with the Moderately Virulent African Swine Fever Virus Strain “Estonia 2014”
Source: Pathogens. 2020 Aug 16;9(8):662. doi: 10.3390/pathogens9080662 (PMC7459997; doi:10.3390/pathogens9080662)
Supplement: Supplementary file 1 [file pathogens-09-00662-s001.pdf]

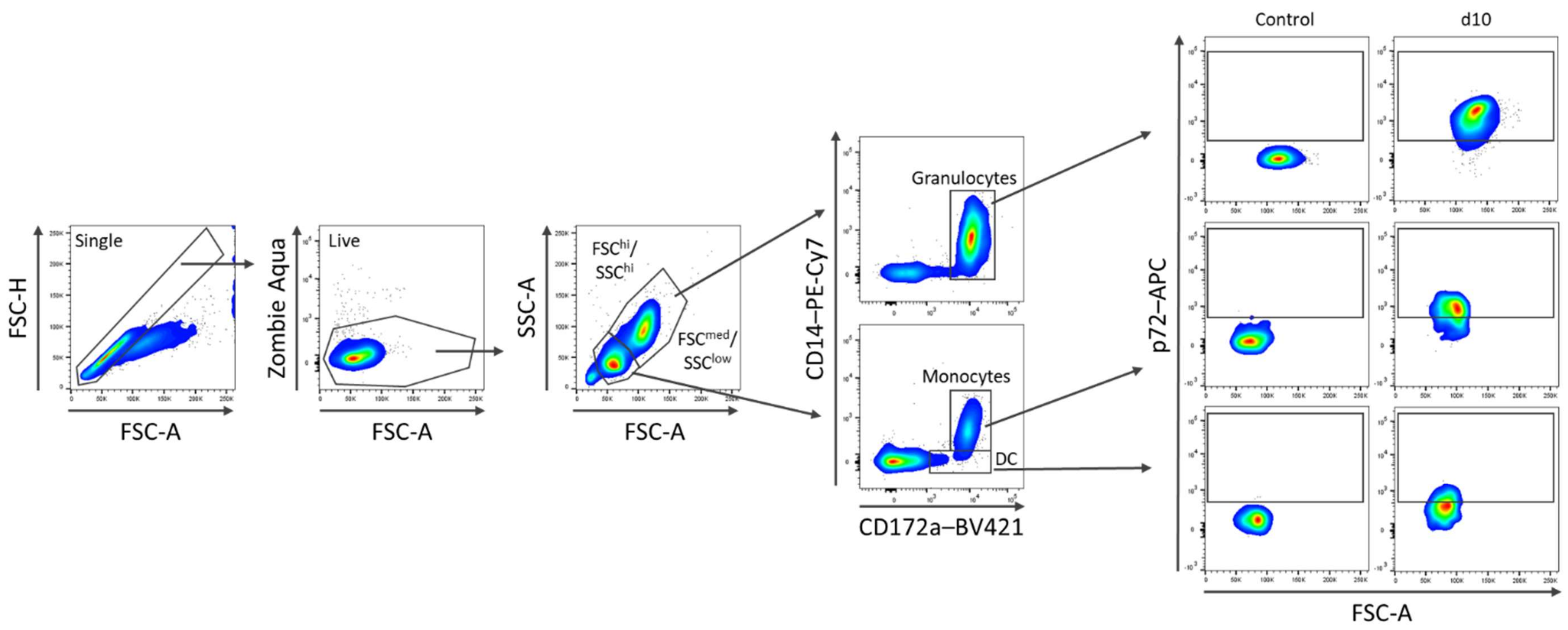

Supplementary figure 1. Gating strategy for the identification of infected myeloid cells by flow cytometry. Single cells were identified by FSC-H vs. FSC-A gating. Subsequently, dead cells were excluded using Zombie Aqua. Single, live cells were further subdivided into FSChi/SSChi and FSCmed/SSClow cells. CD172a+/CD14+ cells among FSChi/SSChi in the blood were identified as granulocytes. In all tissues, monocytes and dendritic cells (DC) were identified as CD172a+/CD14+ and CD172a+/CD14– , respectively, among FSCmed/SSClow cells. p72 expression was analysed of each myeloid cell subset.

Supplementary table 1. Temperature profiles of control and infected domestic pigs from 0 to 10 days pi.

	0 d pi	1 d pi	2 d pi	3 d pi	4 d pi	5 d pi	6 d pi	7 d pi	8 d pi	9 d pi	10 d pi
Co 1	38,9	-	-	-	-	-	-	-	-	-	-
Co 2	38,9	-	-	-	-	-	-	-	-	-	-
DP 30	38,2	38,8	39,3	39,1	38,3	-	-	-	-	-	-
DP 31	39,3	39,2	39,5	39,7	38,7	-	-	-	-	-	-
DP 39	38,3	38,4	39,0	38,4	39,0	-	-	-	-	-	-
DP 35	39,1	38,8	39,3	38,2	38,1	39,3	40,6	40,7	-	-	-
DP 37	39,4	39,2	39,1	39,0	38,5	39,9	41,7	41,0	-	-	-
DP 40	38,8	38,8	39,0	38,9	38,1	39,8	41,2	41,8	-	-	-
DP 32	38,8	39,0	39,1	39,3	39,0	39,7	41,6	41,2	40,9	40,2	39,2
DP 33	38,8	38,5	39,0	38,9	38,5	39,6	40,1	40,9	40,4	40,8	40,6
DP 34	38,6	39,0	38,9	39,2	39,1	39,6	41,1	41,3	40,2	40,2	40,1
